# The Production of Pro-angiogenic VEGF-A Isoforms by Hypoxic Human NK Cells Is Independent of Their TGF-β-Mediated Conversion to an ILC1-Like Phenotype

**DOI:** 10.3389/fimmu.2020.01903

**Published:** 2020-08-25

**Authors:** Lindsey G. Hawke, Mara K. M. Whitford, Mark L. Ormiston

**Affiliations:** ^1^Department of Biomedical and Molecular Sciences, Queen's University, Kingston, ON, Canada; ^2^Department of Medicine, Queen's University, Kingston, ON, Canada

**Keywords:** natural killer cells, transforming growth factor-β, vascular endothelial growth factor-A, angiogenesis, tissue residency

## Abstract

Circulating natural killer (NK) cells have been shown to adopt a type 1 innate lymphoid cell (ILC1)-like phenotype in response to TGF-β and secrete VEGF-A when exposed to hypoxia. Although these changes are often considered to be linked attributes of tissue residency, it has yet to be determined if TGF-β and hypoxia work in concert to coordinate NK cellular phenotype and angiogenic potential. Examination of human circulating NK cells treated with TGF-β demonstrated heterogeneity in their potential to adopt an ILC1-like phenotype, as indicated by the upregulation of CD9 and CD103 on only a subset of cells in culture. Culturing NK cells in chronic hypoxia did not induce a similar ILC1-like conversion and did not enhance the degree of conversion for cells exposed to TGF-β. Similarly, hypoxic culture of circulating NK cells induced VEGF-A secretion, but this production was not enhanced by TGF-β. Fluorescent *in-situ* hybridization flow cytometry demonstrated that hypoxia-induced VEGF-A production was uniform across all NK cells in culture and was not a selective feature of the cellular subset that adopted an ILC1-like phenotype in response to TGF-β. Examination of VEGF-A isoforms demonstrated that hypoxia induces the production of pro-angiogenic VEGF-A isoforms, including VEGF-A_165_ and VEGF-A_121_, and does not stimulate any meaningful production of anti-angiogenic isoforms, such as VEGF-A_b_ transcriptional variants or VEGF-Ax. In summary, TGF-β-mediated ILC1-like conversion and hypoxia-induced VEGF-A production are discrete processes in NK cells and are not part of a linked cellular program associated with tissue residency.

## Introduction

Natural killer (NK) cells have an established role in vascular remodeling. This function is well recognized in pregnancy, where decidual NK (dNK) cells have been linked to an expansion of the spiral arteries that supply oxygen and nutrients to the developing fetus (1–3). Unlike conventional circulating NK cells, dNKs are poorly cytotoxic and produce angiogenic factors, such as vascular endothelial growth factor-A (VEGF-A) (1, 4). dNKs also express a variety of surface markers that are shared by tissue resident NK cells from multiple organs (5), as well as innate lymphoid cell (ILC) subsets like ILC1s (6). Although dNKs, tissue resident NK cells and ILC1s were originally believed to be developmentally distinct from cytotoxic NK cells in the circulation, recent studies have identified significant plasticity in circulating NK cells, whereby they can adopt an ILC1-like phenotype in response to trophoblast or tumor-derived factors, such as transforming growth factor-β (TGF-β) (3, 7–9). This ILC1-like conversion, which is marked by reduced cytotoxicity and acquisition of tissue residency markers, including CD9, CD103, and CD49a, is not equivalent across all NK cells in the circulation, with a distinct subset of this population exhibiting a greater propensity to adopt an ILC1-like phenotype in response to chronic TGF-β exposure (7, 9).

Beyond these changes in cellular phenotype, circulating NK cells can also adopt functions that have previously been linked to dNKs, including the production of VEGF-A. Human circulating NK cells secrete VEGF-A when cultured *ex vivo* under hypoxic conditions (3). An additional study reported VEGF-A production by cultured CD56^+^CD16^−^ NK cells in response to prolonged TGF-β exposure (10). *In vivo*, conditional knockout mice lacking *Vegf* or *Hif1a* specifically in their NK cells have also been shown to exhibit reduced growth of solid tumors, accompanied by impaired tumor vascularization (11, 12).

Together, these studies demonstrate that the angiogenic dNK phenotype may be activated ectopically in tissues outside the uterus in response to tumor-derived factors like hypoxia and TGF-β. Although the implication from these findings is that TGF-β and hypoxia cooperate to induce changes in NK cell phenotype and function, the capacity of these stimuli to converge on shared pathways or cellular programs has not been examined in detail. The precise isoforms of VEGF-A produced by hypoxic NK cells are also uncertain. VEGF-A can exist as either classical, angiogenic isoforms, which promote new vessel sprouting and growth, or anti-angiogenic isoforms, which possess an additional C-terminal domain and may play a role in vessel stability and maturation (13, 14). In the current study, we examine the effect of TGF-β and hypoxia on the NK cell to ILC1-like conversion and VEGF-A production, both alone and in combination. Despite previous suggestions that these stimuli act together to coordinate the conversion of circulating NK cells to an angiogenic ILC1 or dNK-like phenotype, our work demonstrates that TGF-β and hypoxia induce discrete effects that can act independently of each other in a context-dependent manner.

## Method

### NK Cell Isolation and Culture

In accordance with Queen's University Research Ethics Board approval, peripheral blood samples were collected in sodium heparin anti-coagulated vacutainer collection tubes (Greiner BioOne, Kremsmünster, Austria). Peripheral blood mononuclear cells (PBMNCs) were isolated by density gradient centrifugation using Ficoll-Paque (GE Healthcare, Chicago, Illinois) at 500xG for 25 min. The buffy coat was then collected and washed with an equal volume of PBS to remove any Ficoll contamination. NK cells were magnetically isolated from the PBMNCs by negative selection using a NK cell isolation kit (StemCell Technologies, Vancouver, Canada) and cultured in RPMI 1640 (Life Technologies, Carlsbad, California) supplemented with 10% FBS, 50 μM 2-mercaptoethanol and IL-15 (10 ng/mL; Peprotech, Rocky Hill, NJ). Some cultures were supplemented with TGF-β (10 ng/mL; Peprotech) or cultured in hypoxia (1–4% O_2_), as indicated in the figures. Media were refreshed every 4 days on 7–21 day cultures.

### Flow Cytometry

NK cell conversion was assessed by flow cytometry using CD45-Pacific Blue (clone HI30), CD56-PE (clone HCD56), CD3-APC700 (clone UCHT1), CD9-FITC (clone HI9a), and CD103-APC (clone Ber-ACT8), with Zombie NIR dye to assess viability (all from BioLegend, San Diego, California). Intracellular staining for VEGF-A protein was performed using the following antibodies: VEGF-A-APC (clone: 23410, R&D Systems) and VEGF-A (clone: EP1176Y, Abcam). For intracellular assays, cells were cultured for 3 days under various conditions with brefeldin A (1:1,000; BD Biosciences, Franklin Lakes, NJ) added for the final 6 h of the culture. Following staining for viability and surface expression of CD45, CD56, and CD3, samples were fixed, permeabilized, and stained for intracellular VEGF-A. Analysis was performed using FlowJo and cells were gated to only include live, CD45^+^, CD56+, CD3^−^ NK cells.

### VEGF-A Secretion and Isoform Identification by ELISA

NK cell supernatants were collected on days 3, 7, 14, or 21 in culture. VEGF-A secretion was assessed using the human VEGF DuoSet ELISA kit (R&D Systems) as per the manufacturer's protocol. To assess whether NK cells were producing VEGF-A_b_ or VEGFA-Ax isoforms, a custom sandwich ELISA was developed by substituting the VEGF-A_165b_ antibody (clone: 56-1, R&D Systems) in place of the capture antibody used in the original DuoSet ELISA kit.

### Fluorescence *in-situ* Hybridization (FISH) RNA Flow

FISH flow cytometry was used to assess *VEGFA* gene expression in response to chronic hypoxia, with or without TGF-β. Briefly, cells were stained for CD16-PB (clone: 3G8), CD56-PE (clone HCD56), CD3-APC700 (clone UCHT1), CD9-FITC (clone HI9a), and CD103-PECy7 (clone Ber-ACT8) prior to fixation, permeabilization and probing with FISH RNA target probes for *VEGFA* or *B2M* (Invitrogen, Carlsbad, CA) as per the manufacturer's instructions.

### Gene Expression

NK cells were lysed in Trizol (Thermo Fisher, Waltham, MA) after 3 days in culture. RNA was isolated with Direct-zol RNA microprep columns (Zymo Research, Irvine, CA) and reverse transcribed using Superscript IV VILO Mastermix (Invitrogen). Quantitative PCR (qPCR) was performed using PowerUp master mix (Applied Biosystems, Foster City, CA) and primer sets targeting either all *VEGFA* transcripts (pan-*VEGFA*) or specific *VEGFA* splice variants. Pan-*VEGFA* primers were purchased from Fluidigm (San Francisco, CA). *VEGFA*_165*b*+_ primers used a forward primer targeting *VEGFA* exon 7b (CGTACTTGCAGATCTCTACCAGG) and a reverse primer targeting exon 8b (GGTGATGGTGTGGTGGCG). *VEGFA*_121_ and *VEGFA*_165+_ were detected using forward primers targeting exon 5 (GACAAGAAAAATGTGACAAGCCG) and exon 7a (GCAGCTTGAGTTAAACGAACG), respectively, paired with a common reverse primer targeting exon 8a (GGTGATGGTGTGGTGGCG). *VEGFA* gene expression was quantified relative to *B2M* as a housekeeping control, using forward (TTAGCTGTGCTCGCGCTACTCT) and reverse (TGGTTCACACGGCAGGCATACT) primers.

### Immunoblotting

To determine whether the VEGF-A_165b_ antibody (clone: MAB3045; R&D Systems) could also detect the VEGF-Ax isoform, immunoblotting was performed using varying concentrations (0.08–300 ng) of recombinant VEGF-A_165b_ or VEGFA-Ax. Recombinant proteins were run on a 12% SDS-PAGE gel, transferred to polyvinylidene fluoride membranes (Millipore Sigma, St. Louis, MO), blocked with 5% milk in Tris buffered saline containing 0.05% Tween 20 (TBST) and imaged using a Bio-Rad Chemidoc.

### Statistics

Student's *t*-tests were used to compare between two groups. Assessments of multiple comparisons were made by one- or two-way ANOVA with the appropriate *post-hoc* tests. Normal distribution was assumed for all statistical analyses. All statistics used two-sided tests of significance and all data are reported as mean ± SEM.

## Results

### Chronic Hypoxia Does Not Enhance TGF-β-Mediated NK Cell Conversion

**Figure 1:**
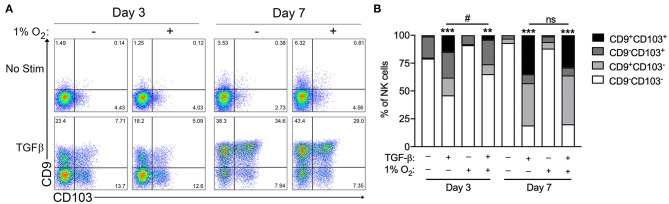
Hypoxia does not enhance TGF-β-mediated NK cell conversion to an ILC1-like phenotype. **(A)** Representative flow cytometry plots showing NK cell acquisition of CD9 and CD103 after 3 and 7 days in culture with or without TGF-β (10 ng/mL) or hypoxia (1% O_2_). **(B)** Summary quantification of NK cell conversion for the conditions detailed in **(A)**. *n* = 3 healthy control subjects, **p < 0.01, ***p < 0.001 relative to unstimulated controls for the corresponding time point. #*p* < 0.05 relative normoxic TGF-β controls for the corresponding time point by two-way ANOVA.

Chronic exposure of cultured circulating NK cells to TGF-β induced their conversion to an ILC1-like phenotype, as indicated by the progressive acquisition of the tissue residency markers CD9 and CD103 (Figure 1A). As reported previously, NK cells exhibited significant heterogeneity in their propensity to convert in response to TGF-β exposure (7). NK cells cultured with TGF-β segregated into distinct single positive (CD9^+^CD103^−^ or CD9^−^CD103^+^), double positive (CD9^+^CD103^+^), or double-negative (CD9^−^CD103^−^) sub-populations, with the proportion cells expressing one or both markers increasing progressively from 3 to 7 days in culture (Figure 1B). In comparison, the exposure of NK cells to chronic hypoxia (1% O_2_) did not induce the upregulation of CD9 or CD103 at either time point. More importantly, the combination of chronic hypoxia and TGF-β did not enhance NK cell conversion over 7 days, when compared to TGF-β alone. Instead, hypoxic exposure decreased the rate of TGF-β-mediated NK cell conversion, resulting in a significantly reduced proportion of converted cells after 3 days.

### TGF-β Does Not Enhance Hypoxia-Induced VEGF-A Production

**Figure 2:**
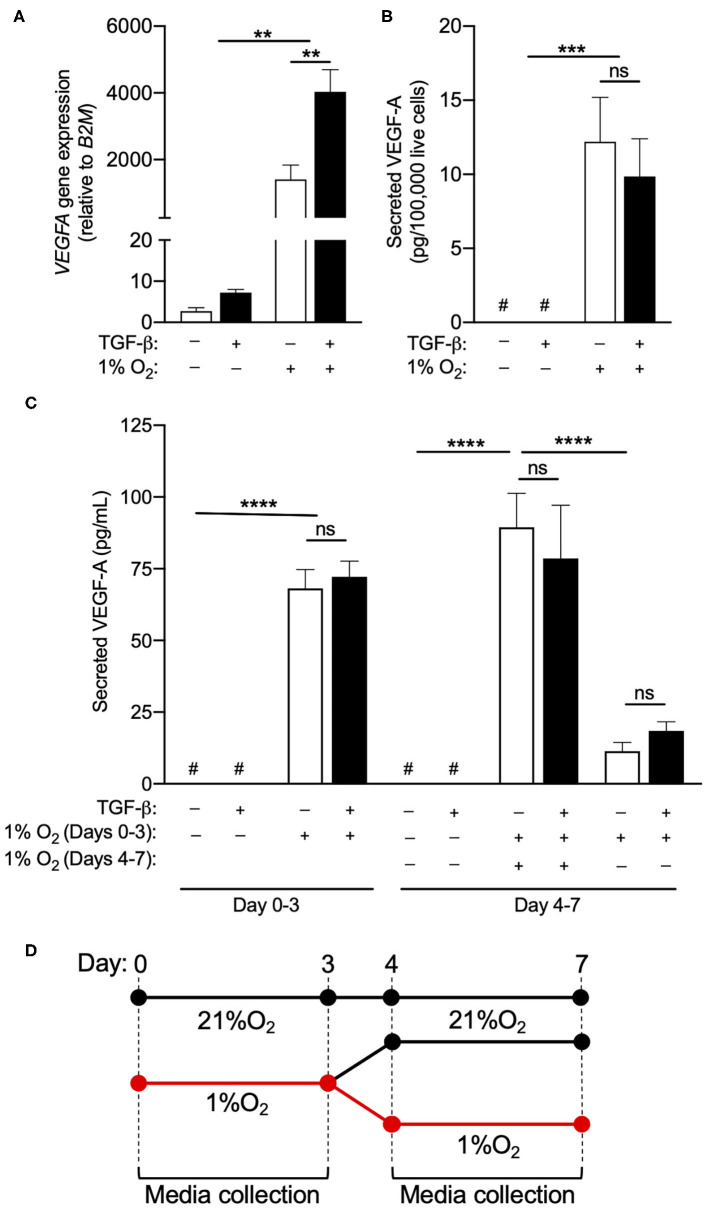
TGF-β does not enhance hypoxia-induced VEGF-A production. **(A)** *VEGFA* gene expression relative to daily normoxic basal media after 3 days in culture with or without TGF-β (10 ng/mL) or hypoxia (1% O_2_). **(B)** Secreted VEGF-A per 100,000 live cells after 3 days in culture with or without TGF-β (10 ng/mL) and hypoxia (1% O_2_). **(C)** Absolute secreted VEGF-A after 3 or 7 days with or without TGF-β (10 ng/mL) and hypoxia (1% O_2_). **(D)** Schematic depiction of the culture conditions in **(C)**. After 3 days in hypoxia, a subset of NK cells were returned to normoxia for 4 days to observe whether VEGF-A production persists when cells re-enter the normoxic state. **p < 0.01, ***p < 0.001, ****p < 0.0001 by two-way ANOVA with Sidak's *post-hoc* test to determine effects of TGF-β. ^#^Indicates measurement below detection limit of ELISA.

Although hypoxia did not enhance the acquisition of an ILC1-like phenotype, the exposure of circulating human NK cells to 1% O_2_ for 72 h did cause a nearly 2,000-fold increase in *VEGFA* gene expression (Figure 2A). TGF-β treatment further enhanced *VEGFA* mRNA levels in NK cells exposed to chronic hypoxia, suggesting that the NK to ILC1-like conversion may be associated with an elevation in the angiogenic capacity of these cells. Assessment of culture supernatants confirmed the induction of VEGF-A secretion by hypoxic NK cells, when compared to normoxic control samples that were below the detectable limit of the ELISA (Figures 2B,C). However, in contrast to the findings for *VEGFA* gene expression, TGF-β treatment did not induce the secretion of VEGF-A protein in normoxia and failed to enhance the production of VEGF-A by NK cells exposed to hypoxia. Importantly, TGF-β did not show any effect on VEGF-A secretion when quantified on a per-cell basis (Figure 2B) or when presented as an absolute concentration (Figure 2C), indicating that the discord between *VEGFA* gene expression and protein production was not the result of changes in the number of viable NK cells in each culture condition. Assessment of extended cultures also failed to detect an induction of VEGF-A production by NK cells treated with TGF-β for 7, 14, or 21 days (Figure 2C; data not shown).

In order to determine if VEGF-A production was a transient phenomenon or the consequence of a permanent reprogramming of NK cell phenotype, NK cells exposed to hypoxia for 3 days were returned to a normoxic environment for an additional 4 days, with VEGF-A secretion measured over the final 72 h of this period (Figure 2D). Under these conditions, VEGF-A production by hypoxic NK cells returned to near baseline in normoxia (Figure 2C), indicating this response is not linked to a permanent change in cellular phenotype.

### VEGFA Expression Is Not Enhanced by ILC1-Like Phenotype Conversion

**Figure 3:**
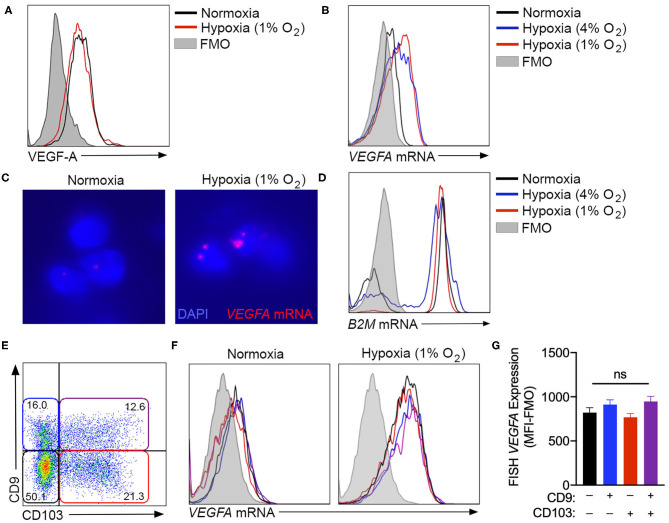
*VEGFA* expression is not enhanced by ILC1-like phenotype conversion. **(A)** Unsuccessful detection of intracellular VEGF-A production by flow cytometry using the anti-VEGF-A clone 23410. Cells were cultured in with or without hypoxia (1% O_2_) for 3 days. **(B)** Representative fluorescence *in-situ* hybridization (FISH) flow cytometry plot quantifying *VEGFA* gene expression by NK cells cultured in normoxia (black) or hypoxia at 4% O_2_ (blue) or 1% O_2_ (red). **(C)** Fluorescent microscopy detection of FISH *VEGFA* mRNA probes (red) in normoxic or hypoxic (1% O_2_) NK cells counterstained with DAPI (blue). **(D)** Representative FISH flow cytometry for *B2M* control gene expression under the culture conditions detailed in **(B)**. **(E)** Representative flow cytometry plot of CD9 and CD103 expression after 3 days in culture with TGF-β and hypoxia, demonstrating gating for CD9^−^CD103^−^ (black), CD9^+^CD103^−^ (blue), CD9^−^CD103^+^ (red), and CD9^+^CD103^+^ (purple) NK cell subsets. **(F)** Representative flow cytometry histograms demonstrating FISH-detected *VEGFA* expression under normoxia and hypoxia for the subsets gated in **(E)**. **(G)** Quantification of FISH-*VEGFA* expression for the subsets gated in **(E)** on NK cells cultured for 3 days with TGF-β and hypoxia (1% O_2_). (MFI-FMO = median fluorescence intensity less fluorescence minus one control). *n* = 3. ns = not significant by ANOVA.

While the assessment of tissue culture supernatants by ELISA was suitable to quantify bulk VEGF-A production by cultured NK cells, intracellular flow cytometry was used to determine if this hypoxic response was equivalent across all cells in culture, or was selectively upregulated by specific subsets within the heterogeneous population. However, multiple monoclonal antibodies, including clones 23410 (R&D Systems) (Figure 3A) and EY1176Y (Abcam, data not shown), failed to demonstrate any increase in VEGF-A production with chronic hypoxia. As an alternative, fluorescence *in-situ* hybridization (FISH)-flow cytometry was employed to quantify *VEGFA* mRNA expression at the single-cell level. This technique confirmed an increase in *VEGFA* mRNA in NK cells exposed to 72 h of either 4 or 1% oxygen, when compared to normoxic controls (Figures 3B,C). Importantly, expression of the *B2M* housekeeping gene remained consistent across all culture conditions when assessed using this technique (Figure 3D).

Using FISH-flow, NK cells cultured in the presence of TGF-β, with or without chronic hypoxia, were examined to determine if *VEGFA* gene expression was selectively elevated in the cellular subset that exhibited TGF-β-induced ILC1-like conversion (Figures 3E,F). These studies demonstrated an equivalent degree of *VEGFA* mRNA in both unconverted cells and those that acquired CD9, CD103, or both markers in the presence of both TGF-β and hypoxia (Figure 3G). Together, these findings indicate that hypoxia-induced *VEGFA* expression is not a selective feature of the subset of circulating NK cells that most readily adopt a tissue resident phenotype in response to TGF-β.

### Circulating NK Cells Produce Pro-angiogenic VEGF-A Isoforms

**Figure 4:**
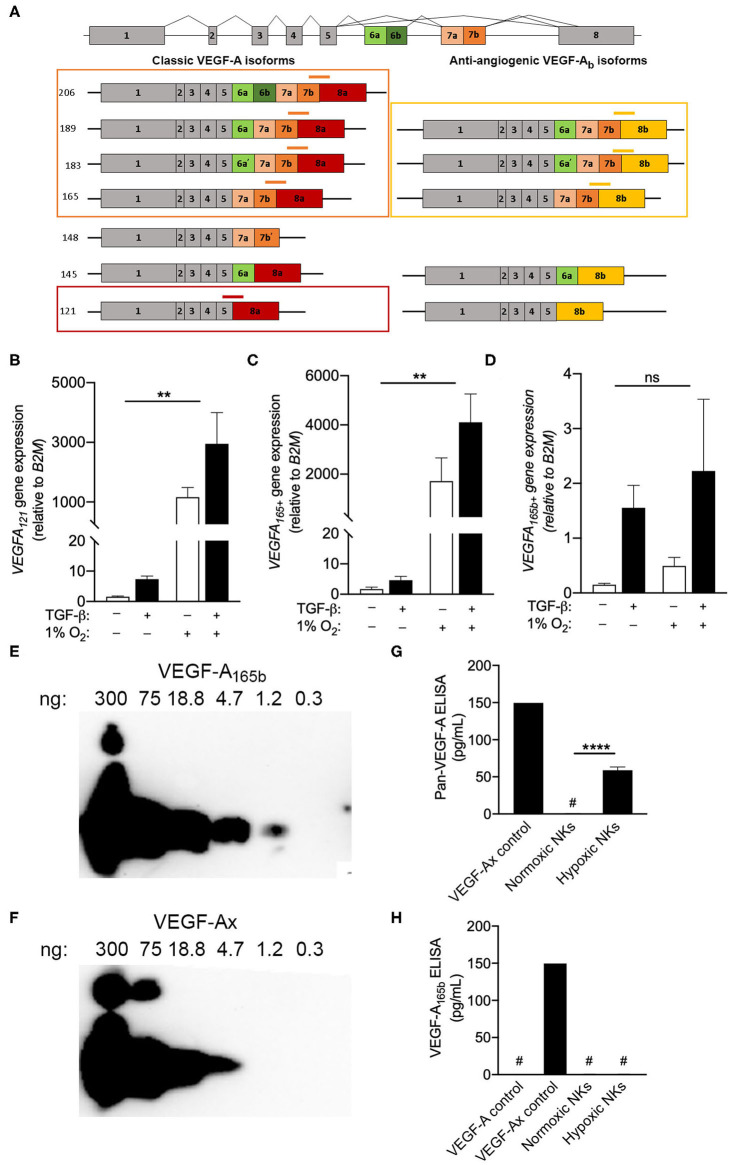
Circulating NK cells produce pro-angiogenic VEGF-A isoforms. **(A)** Schematic of *VEGFA* alternative splicing of classic angiogenic VEGF-A isoforms and anti-angiogenic VEGF-A_b_ isoforms. Custom exon spanning primers for *VEGFA*_121_ (red), *VEGFA*_165,183,189,206_ (*VEGFA*_165+_, orange), and *VEGFA*_165*b*+_ (yellow) splice variants are shown. **(B–D)** Expression of **(B)** *VEGFA*_121_, **(C)** *VEGFA*_165+_, and **(D)** *VEGFA*_165*b*+_ splice variants after 3 days in culture with or without TGF-β and or hypoxia (1% O_2_) relative to normoxic controls. *N* = 4, **p < 0.01 by two-way ANOVA with Sidak's *post-hoc* test to determine effects of TGF-β (all non-significant). **(E,F)** Representative immunoblots demonstrating detection of **(E)** VEGF-A_165b_ and **(F)** VEGF-Ax using the anti-VEGF-A_165b_ clone 56-1. **(G,H)** Quantification of **(G)** all VEGF-A isoforms and **(H)** anti-angiogenic VEGF-A isoforms (VEGF-A_165b_ or VEGF-Ax) in day 3 NK cell culture supernatants by sandwich ELISA. *N* = 4–6, ****p < 0.0001 by Student's *t*-test. ^#^Indicates measurement below detection limit of ELISA.

The inability of multiple monoclonal antibodies to detect VEGF-A production by intracellular flow cytometry drew attention to the C-terminal region of the protein, which contains the epitopes for all antibodies tested. Interest in this region is particularly relevant when considering the fact that VEGF-A is known to exist as both angiogenic and anti-angiogenic isoforms, which are defined by the exclusion or inclusion, respectively, of an extended C-terminal domain (15). Importantly, anti-angiogenic VEGF-A isoforms, which can be produced either through alternative splicing (15–17) or via the modified translation of conventional transcripts (14), would not be distinguished from angiogenic isoforms by the qPCR, FISH-flow or ELISA methods used in Figure 3. In order to determine the precise *VEGFA* transcripts produced by NK cells exposed to chronic hypoxia, custom primers were designed to target specific *VEGFA* splice variants (Figure 4A). Selective primers for classical pro-angiogenic *VEGFA* transcripts, including those encoding VEGF-A_121_ or longer forms like VEGF-A_165/183/189/206_ (*VEGFA*_165+_), demonstrated a robust upregulation of these transcripts under hypoxic conditions (Figures 4B,C) that was in-line with the increase observed using non-selective *VEGFA* primers (Figure 2A). In contrast, primers for anti-angiogenic VEGF-A_b_ isoforms, which include exon 8b of the *VEGFA* gene at the 3' end of the mRNA coding region, demonstrated no response to hypoxia and only modest, statistically insignificant changes with TGF-β that were several orders of magnitude smaller than those seen for the pro-angiogenic transcripts (Figure 4D). As such, these anti-angiogenic transcripts are unlikely to be a major contributor to the VEGF-A produced by NK cells.

Beyond alternative splicing, previous studies have also reported the production of an additional anti-angiogenic VEGF-A isoform, VEGF-Ax, which is produced as a C-terminal translational variant of classical *VEGFA* transcripts. A monoclonal antibody for VEGF-A_165b_ (clone 56-1) was examined for its ability to detect of VEGFA-Ax, which shares seven C-terminal amino acids with VEGF-A_165b_ (14). Immunoblotting demonstrated the capacity of clone 56-1 to detect recombinant VEGFA-Ax, albeit with reduced sensitivity when compared to VEGF-A_165b_ (Figures 4E,F). For the assessment NK culture supernatants, a pan-VEGF-A ELISA was modified to detect VEGF-Ax, but not conventional VEGF-A isoforms, by substituting in clone 56-1 as the detection antibody (Figures 4G,H). Assessment of tissue culture supernatants using the pan-VEGF-A ELISA confirmed the production of VEGF-A by NK cells exposed to hypoxia. However, the modified ELISA failed to detect any VEGF-A_165b_ or VEGF-Ax in these supernatants, indicating that NK cells produce exclusively pro-angiogenic VEGF-A isoforms under hypoxic conditions.

## Discussion

Our work demonstrates that TGF-β and hypoxia exert independent effects on circulating NK cells. TGF-β drives the acquisition of a tissue residency or ILC1-like phenotype, including the expression of surface markers like CD9 and CD103, but this process is not enhanced by exposure to chronic hypoxia. Meanwhile, the capacity of NK cells to produce VEGF-A is regulated by exposure to hypoxia but is not linked with NK cell conversion to an ILC1-like phenotype. Interestingly, TGF-β enhances *VEGFA* gene expression in hypoxic NK cells, but does not alter VEGF-A protein secretion. This observation raises the possibility that additional factors present in tissues, such as IL-18 and IL-12, may be required to induce the translation of these pre-formed transcripts and increase VEGF-A protein production. Even long-term exposure of circulating NK cells to TGF-β for 7-21 days failed to induce measurable VEGF-A secretion, contrasting with previous reports that used intracellular flow cytometry to quantify VEGF-A production (10). These findings argue against the suggestion that TGF-β and hypoxia act in concert to promote the conversion of circulating NK cells to an angiogenic dNK-like phenotype (3). Importantly, our work also shows that VEGF-A production by circulating NK cells is a transient phenomenon, which can be reversed upon returning hypoxic NK cells to a normoxic environment. This result demonstrates that at least some of the cellular plasticity observed in circulating NK cells is bidirectional and is not associated with a permanent change in cellular function.

While the effects of TGF-β and hypoxia on NK cells are independent, it is still probable that both factors are required *in vivo* to illicit NK-mediated changes in vascular structure. Previous works have shown that TGF-β, in combination with other factors like IL-15, induce the expression of vital integrins, like CD9 and CD103, not only on NK cells, but also on other lymphocyte subsets like resident memory T cells (7, 18, 19). Such changes are essential to the migration and retention of these lymphocyte subsets into tissues (19). Upon exiting the circulation, local exposure of NK cells to a reduced oxygen environment would allow for VEGF-A production and the potential to influence localized vascular remodeling. Importantly, our work with FISH-flow cytometry demonstrates that even 4% O_2_, which is equivalent to normoxia in many tissues (20), induces an upregulation of *VEGFA* gene expression in NK cells that is similar in magnitude to what is observed with 1% O_2_. Therefore, severely hypoxic environments, such as poorly vascularized solid tumors, are not the only *in vivo* settings where NK cells can contribute to angiogenic activity.

Our quantification of *VEGFA* expression on a single-cell level using FISH-flow cytometry also demonstrated that *VEGFA* is upregulated uniformly across all NK cells in hypoxic cultures and is not attributable to a specialized subset of circulating NK cells. Co-staining of *VEGFA* mRNA with CD9 and CD103 in hypoxic NK cells revealed no enrichment of *VEGFA* gene expression in the subset of NK cells that adopt an ILC1-like phenotype in response to TGF-β vs. those that did not, further supporting the conclusion that the actions of TGF-β are independent of the angiogenic functions of hypoxic NK cells. Importantly, the use of FISH-flow cytometry for this purpose was necessitated by the failure of multiple antibodies to detect VEGF-A using standard intracellular staining protocols. This result, which contrasts with other publications that used the same anti-VEGF-A antibodies for this purpose (10, 21–24), highlights the need for robust positive and negative controls in such assays. Moving forward, future studies may make use of FISH-flow cytometry to evaluate *VEGFA* expression among tissue-resident or tumor-infiltrating NK and ILC1 cells, with the goal of determining if there is an *ex vivo* association between *VEGFA* expression and the TGF-β-associated ILC1-like phenotype.

Our examination of the VEGF-A isoforms produced by hypoxic NK cells determined that these cells produce exclusively classical, pro-angiogenic isoforms of VEGF-A, such as VEGF-A_165_ and VEGF-A_121_. In contrast, anti-angiogenic VEGF-A isoforms containing alternative C-terminal domains, such as VEGF-A_b_ transcriptional variants or the transcriptional read-through product, VEGF-Ax, were not significantly upregulated by hypoxia. The anti-angiogenic functions of these isoforms have been suggested to contribute to the stabilization and maturation of newly formed vessels through their differing interactions with the VEGF co-receptor neuropilin-1 and heparin/heparan sulfate proteoglycans, as well as by acting as a competitor for classical VEGF-A signaling through VEGFR1 and VEGFR2 (15, 25–27). Production of such variants by hypoxic NK cells could have explained the leaky, disorganized vasculature observed in the tumors of mice bearing an NK-conditional deletion of Hif-1α (11). Although our work indicates that NK cells do not produce these forms of VEGF-A, it is possible that hypoxia causes NK cells secrete other factors that can contribute to vessel maturation and stability *in vivo*.

In summary, the current study has provided insight into how factors like hypoxia and TGF-β regulate the capacity of NK cells to flux between their circulating, cytotoxic phenotype, and a more cytokine producing, angiogenic functionality. These studies have potential implications for understanding the contribution of NK cells to vascular remodeling in pregnancy, as well as their role in the vascularization of solid tumors.

## Data Availability Statement

The raw data supporting the conclusions of this article will be made available by the authors, without undue reservation. Requests to access the datasets should be directed to mark.ormiston@queensu.ca.

## Ethics Statement

The studies involving human participants were reviewed and approved by Queen's University Research Ethics Board. The patients/participants provided their written informed consent to participate in this study.

## Author Contributions

LH designed and performed experiments, analyzed data, and wrote manuscript. MW designed and performed experiments and analyzed data. MO conceived study, designed experiments, and wrote manuscript. All authors contributed to the article and approved the submitted version.

## Conflict of Interest

The authors declare that the research was conducted in the absence of any commercial or financial relationships that could be construed as a potential conflict of interest.
